# Outcomes of paediatric patients with chronic liver disease in early adulthood: A heterogeneous, but representative, regional cohort study

**DOI:** 10.1111/jpc.16091

**Published:** 2022-06-27

**Authors:** Nicole SM Lau, Paul Henderson

**Affiliations:** ^1^ Child Life and Health University of Edinburgh Edinburgh United Kingdom; ^2^ Department of Paediatric Gastroenterology and Nutrition Royal Hospital for Children and Young People Edinburgh United Kingdom

**Keywords:** epidemiology, follow‐up studies, liver diseases, longitudinal studies, paediatrics

## Abstract

**Aim:**

Advances in paediatric hepatology have led to the increasing survival of patients with paediatric‐onset chronic hepatobiliary disease into adulthood. Data are lacking with regard to the outcomes of this heterogeneous group of patients and current transition models may be insufficient. This retrospective regional cohort study examined the outcomes of these patients cared for in a paediatric gastroenterology centre following transfer to adult services.

**Methods:**

A prospective database of paediatric patients with liver disease identified those already transferred to adult services. Following exclusions, medical notes were examined and health parameters recorded including initial diagnoses, transplant status, fertility and mortality. Descriptive statistics were used to calculate follow‐up data and transplant‐free survival (TFS).

**Results:**

Overall, 63 patients (52% male) entered the final analyses with a median follow‐up of 27.5 years. The most common diagnosis was biliary atresia (19%); 27 different diagnoses were apparent within the cohort highlighting the heterogeneity within a single centre. Transplant prevalence at adult transfer was 41%; 14% of patients were lost to follow‐up including 10% of transplant patients. TFS for biliary atresia was 17% after 37.4 years follow‐up and was 54% for the total cohort. There were seven documented pregnancies and the prevalence of any psychological or psychiatric input was 44%. Transplant complications occurred in 38% of patients; there were two cancer diagnoses and two deaths following transfer.

**Conclusions:**

Although overall mortality was low, the health‐care burden of patients with paediatric‐onset chronic liver disease is high. This group is also very heterogeneous, making structured transition to adult services difficult.

## What is already known on this topic

1. The long‐term outcomes of children following liver transplantation and with specific conditions are largely known.
2. The heterogeneity and outcomes of population‐based cohorts of children with chronic liver disease transitioning to adult centres from their local gastroenterology unit are unknown.

## What this paper adds

1. Children with chronic liver diseases have significant morbidity in early adulthood and their overall health‐care burden remains high.
2. Children with chronic liver diseases represent a very heterogeneous group and may require condition‐specific transition models.

Advances in paediatric hepatology throughout the 1980s and 1990s have led to the increasing survival of patients with paediatric‐onset chronic hepatobiliary disease into adulthood.^1^, ^2^ Diseases once unique to the paediatric setting are now seen in adult practice.^3^ Although the structured transition from paediatric to adult services is now commonplace for chronic liver disease,^4^, ^5^ standards have only recently been established; therefore, health outcomes in early adulthood may not be affected for some time. There is a paucity of basic health data available regarding this group in early adulthood, with previous studies often focussing on single conditions, those requiring transplant only, or using historical cohorts.^6^, ^7^, ^8^, ^9^, ^10^ Although the incidence of chronic paediatric liver diseases is low overall, this group of patients rely heavily on health‐care services when considered together.

This retrospective regional cohort study aimed to assess the basic health outcomes of patients with paediatric‐onset chronic liver disease from a tertiary paediatric gastroenterology centre following their move to adult services. Given the lack of published guidance during the study period, a focus on the transition process itself was deliberately avoided. Overall, consisting of a relatively small and heterogeneous group of patients, this cohort was felt to in fact be *more* representative of the majority of centres transitioning this patient group, without the risk of transplant centre bias.^5^, ^11^

## Materials and Methods

### Setting

Within Scotland (and throughout the United Kingdom (UK)), all tertiary paediatric gastroenterology units are loosely affiliated with one of the three quaternary paediatric liver transplant centres (London, Birmingham and Leeds). Centralisation of services occurred in the early 2000s and although a specialist paediatric hepatologist will often attend satellite clinics within these tertiary centres to help monitor these patients: (i) the day‐to‐day care of these patients remains under the direct guidance of the tertiary gastroenterologist; (ii) some patients may never be known to, or have a face‐to‐face meeting with, the quaternary specialist; and (iii) the majority of adolescent patients will transition to the care of an adult gastroenterologist/hepatologist locally with no input from quaternary centres.

The Department of Paediatric Gastroenterology and Nutrition at the Royal Hospital for Sick Children, Edinburgh (RHSCE; recently renamed Royal Hospital for Children and Young People) was established in August 1997. The hospital has always acted as a referral centre for all hospitals in South‐East Scotland (SES). RHSCE is not, and never has been, a paediatric liver transplant centre. With regard to adult transition, this was performed *ad hoc* with the local adult hepatology unit from 1997 to 2001 with more straightforward patients transferred by way of a single transfer letter. Since 2002, a more formal transition process has been adopted with a single clinic, attended by a paediatric gastroenterologist, adult hepatologist and specialist nurses, held in RHSCE each spring followed by an identically structured MDT clinic held in the local adult hepatology centre in the autumn. Due to the time frame of this study, the process of transition to adult services has changed as new evidence became available. In the UK, patients would often remain in paediatrics until they leave secondary education (around 17–18 years of age), but a proportion of patients transition earlier due to several factors. Throughout the study period, assessment readiness for transition using a formal tool was not used; however, nurse specialists would meet the young person separately around the time of transition to discuss drug compliance, understanding of medications and their side effects, the disease itself and other issues relating to sexuality, education, smoking and alcohol. In a small number of cases, a single transfer letter still remains the only feasible mechanism of paediatric to adult transfer.

### Patient cohort

Since 1st August 1997, RHSCE has been prospectively collecting data on paediatric patients with chronic liver disease and now holds details of over 350 patients. From this database, patients 16 years and over by 31st December 2018 (hereafter referred to as ‘study end’) were identified and excluded if they: (i) had moved out of the region while still in paediatric care; (ii) died while in paediatric services; (iii) were discharged at or before transition/transfer to adult services; (iv) were still under the care of paediatric services at study end; or (v) were living outside of the SES region as described above based on postcode at study end. Accurate incidence and prevalence data for all paediatric chronic liver diseases are not available either in the UK or elsewhere.

### Data collection

Clinical data were collected from electronically stored letters and the TrakCare Electronic Medical Record System (InterSystems Corporation, Cambridge, MA, USA); data were then collated in Microsoft Excel 2007 (Microsoft Corporation, Redmond, WA, USA). Due to the historical nature of some medical records, the precise date of diagnosis was not always available. In these cases, the following criteria were used as the date of diagnosis: (i) first mention of the confirmed paediatric liver disease diagnosis in correspondence; (ii) the date of Kasai procedure for patients with biliary atresia; (iii) for hepatitis B and C patients their oldest correspondence with mention of their diagnosis. (The social circumstances of many patients with hepatitis B and C meant that follow‐up was often sporadic.) Transfer was defined as patients who had moved directly from paediatric to adult services, with a single transfer letter, with the date of transfer being the last paediatric clinic appointment. Transition was defined as the formal process described above with the transition date taken as the first joint paediatric and adult transition clinic appointment. Patients were classed as lost to follow‐up if there were at least three non‐attendances to clinic and/or no tertiary health‐care interaction within the past 3 years by study end. Patients discharged to the care of their community general practitioner while in paediatric services and patients under the care of the department of Inherited and Metabolic Diseases (who transition patients independently) were excluded. All patients (if compliant with follow‐up arrangements) have regular bloods (including alpha‐fetoprotein) and imaging as part of their routine follow‐up, especially with regard to hepatocellular carcinoma screening.

### Statistics

Descriptive statistics of the cohort were calculated using Microsoft Excel (Microsoft Corporation, Redmond, WA, USA). Median and interquartile range (IQR) were used for non‐parametric variables. Transplant‐free survival (TFS) rates were calculated at the point of adult transfer and also calculated at the point of last known follow‐up or study end, whichever was latest. TFS in adult services ended at liver transplantation, study end or death, whichever was latest.

### Ethics

Clarification with the South‐East Scotland Ethics Scientific Officer established that formal ethical approval was not required for this type of retrospective study. However, a Children's Services' project proposal and registration form were approved by the RHSCE Quality Improvement Team. This study conforms to the provisions of the Declaration of Helsinki.

## Results

### Patient cohort

**Fig. 1:**
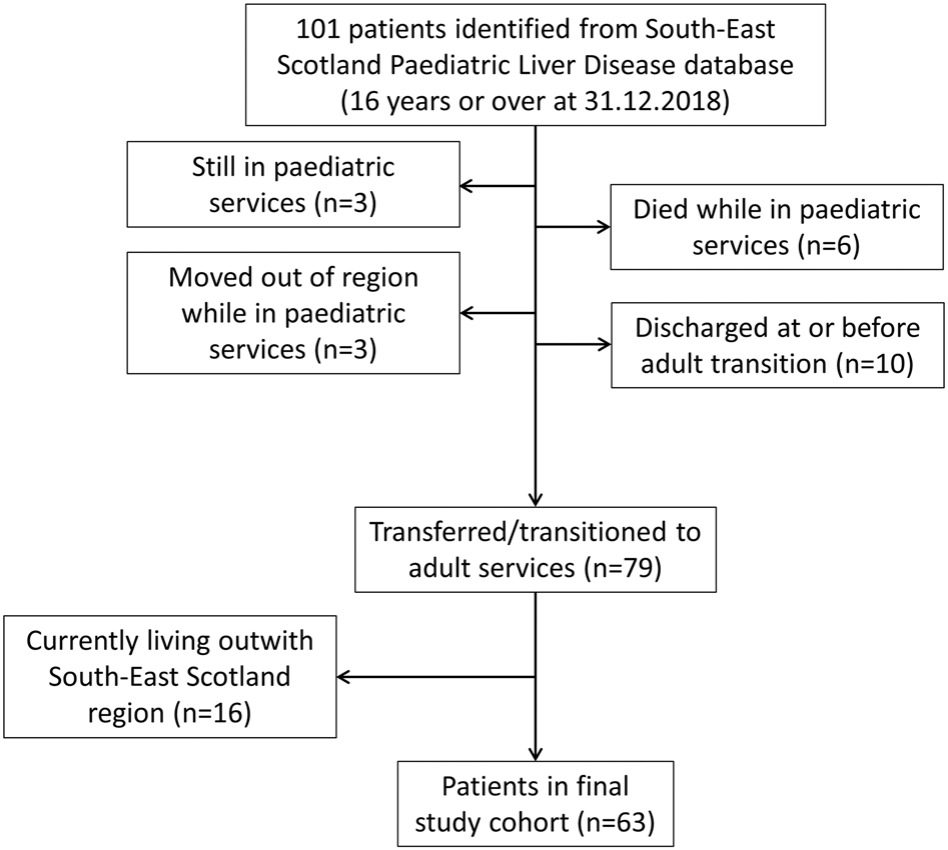
Flow diagram describing the derivation of the patient cohort.

**Fig. 2:**
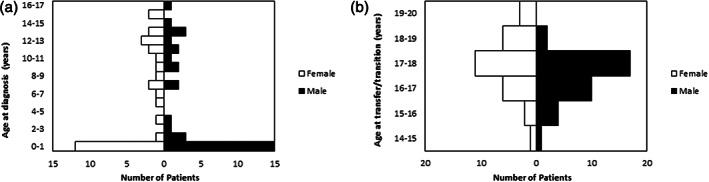
(a) Population pyramid demonstrating age at diagnosis and sex distribution; (b) Population pyramid demonstrating age at transfer/transition and sex distribution.

Initial screening identified 101 patients to be 16 years and over at study end; 63 patients entered the final analyses (Fig. 1). The cohort consisted of 33 males and 30 females with the distribution of age and sex at liver disease diagnosis shown in Figure 2A; median age was 2.8 years (IQR 0.1–11.5). Of note, 27 patients (43% of the cohort) were aged less than 1 year old at diagnosis.

**Fig. 3:**
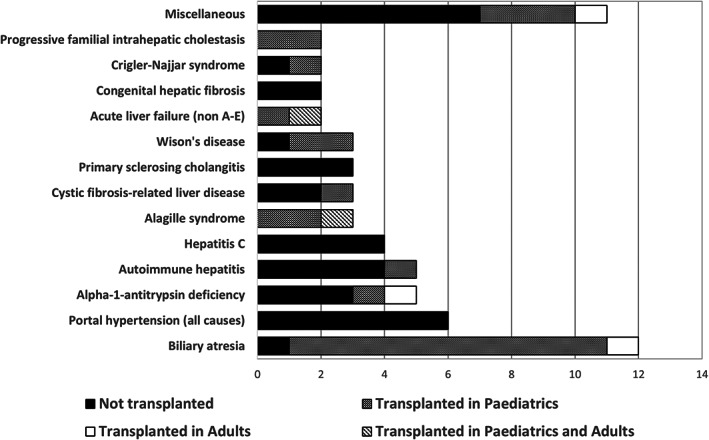
Diagnoses and transplant status in the cohort of paediatric patients with chronic liver disease followed into adulthood.

Final diagnoses of the cohort are shown in Figure 3. The most common diagnosis was biliary atresia (*n* = 12; 19% of the cohort). The six patients with portal hypertension had the following diagnoses: cavernous transformation of the portal vein (*n* = 2); idiopathic portal vein thrombosis (*n* = 2) and hepatoportal sclerosis secondary to thiopurines following treatment for acute lymphoblastic leukaemia (*n* = 2). There was one patient with progressive familial intra‐hepatic cholestasis type 1 (no genetic confirmation available; diagnosed in 1992) and one patient with BSEP deficiency (confirmed mutation in *ABCB11*). Further highlighting the rarity of most chronic paediatric liver diseases, the miscellaneous group consisted of 11 patients with unique diagnoses within the cohort: autoimmune sclerosing cholangitis, blunt liver trauma, choledochal cyst, congenital biliary hypoplasia, focal nodular hyperplasia with a congenital portocaval shunt, hepatitis B, hepatoblastoma, intestinal failure‐associated liver disease, Jeune syndrome, non‐alcoholic fatty liver disease (NAFLD) and unilateral naevoid telangiectasia.

While in paediatric services, there were a total of 29 transplants performed in 26 patients (Fig. 3). Median time to transplant following diagnosis in paediatric services was 3.8 years (IQR 0.6–8.5). Transplant prevalence at transition/transfer was 41% and TFS of the cohort at transition/transfer was 59% (37 of 63).

Overall, 14 patients were transferred and 49 underwent formal transition at a median age of 17.2 years (IQR 16.5–17.3; Fig. 2B). The median length of follow‐up in paediatric services was 14.0 years (IQR 6.0–18.0).

### Basic health outcomes while in adult services

#### Follow‐up

The median age of the entire cohort was 26.3 years (range 16.2–36.9, IQR 20.7–29.8) at study end. Following transition/transfer to adult services, seven patients moved out of the region and nine were lost to follow‐up. Notably, no patient was formally discharged from follow‐up for their liver disease. The combined follow‐up patient‐years from diagnosis was 1753 patient‐years with median follow‐up per patient of 27.5 years (IQR 19.5–37.3). For those lost to follow‐up, median time in adult services was 4.5 years (IQR 2.9–11.1). The diagnoses in those patients who were lost to follow‐up were hepatitis C (*n* = 3), biliary atresia (*n* = 2), alpha‐1‐antitrypsin deficiency (*n* = 3) and idiopathic portal hypertension (*n* = 1). Importantly, among these, three of the patients had undergone liver transplant at transition (two had biliary atresia and one had alpha‐1‐antitrypsin deficiency); therefore, overall 10% of transplant patients were lost to follow‐up in tertiary services. However, these patients may have remained under the care of their community general practitioner.

#### TFS, fertility, mental health and cancer

The TFS for the cohort at study end was 54% (34 of 63). The TFS for patients with biliary atresia was 17% (2 of 12) at a median of 37.4 years (IQR 33.2–42.0) from diagnosis. There were six patients with nine documented pregnancies (20% of females) during follow‐up. A summary of these patients and their pregnancies are shown in Table 1. It was not possible to collect data on the partners of male subjects with regard to male fertility. Twenty‐eight patients had at least one documented consultation within either psychology or psychiatric services during follow‐up (44% of cohort); details of psychological and psychiatric diagnoses were not available. Only two patients had a cancer diagnosis following transfer to adult services: acute myeloid leukaemia and hepatocellular cancer, both following transplantation.

**Table 1:** Pregnancy outcomes in paediatric patients with liver disease following transfer/transition to adult services

Primary diagnosis	Transplant^†^	Gravidity at end of follow‐up	Complications during pregnancy	Pregnancy summary
Alagille Syndrome	P	G3 P1 + 2	Sepsis from dental abscess, mild acute rejection	1 liveborn (with Trisomy 21), 1 miscarriage
				Before 24 weeks and 1 medical termination at 12 weeks
Hepatitis C		G1 P0 + 1		1 miscarriage before 24 weeks
PSC^‡^		G1 P1	Small bowel obstruction due to gravid uterus	1 liveborn
Biliary Atresia	P	G1 P0 + 1		1 medical termination before 24 weeks
A1AT^§^ deficiency	A	G2 P2	Sepsis, pre‐eclampsia, acute kidney injury, deferred repeat liver transplant, thrombosis	1 liveborn and 1 neonatal death at 24 weeks
PFIC¶	P	G1 P0		Still pregnant at study end

^†^ P = Paediatrics; A = Adults.

^‡^ Primary sclerosing cholangitis.

§ Alpha‐1‐antitrypsin.

¶ Progressive familial intrahepatic cholestasis.

#### Liver transplant and mortality

Within the cohort, two patients underwent re‐transplant in adult services; one patient with an initial diagnosis of fulminant liver failure of unknown cause developed liver cirrhosis and hepatocellular carcinoma and a patient with Alagille syndrome developed acute‐on‐chronic rejection while on mycophenolate mofetil. Of the three patients that had their first liver transplant in adult services, their diagnoses were biliary atresia, congenital biliary hypoplasia and alpha‐1‐antitrypsin deficiency (Fig. 3). Their first transplant occurred at a median of 13.2 years (IQR 10.9–14.0) following transfer/transition to adult services. Therefore, from initial diagnosis, the median time to first transplant for the entire cohort was 5.1 years (IQR 0.7–10.7). Of the total 29 patients who had undergone transplantation by the end of follow‐up, 11 patients (38%) experienced significant transplant‐related complications. Complications are shown in Table 2.

**Table 2:** Transplant‐related complications in paediatric patients with liver disease following transfer/transition to adult services

Complication	No. of patients†
Portal hypertension (i.e. oesophageal varices; no acute bleeding)	**4**
Chronic rejection	**2**
Acute rejection	**2**
Hepatocellular carcinoma in graft	**1**
Portal vein thrombosis	**1**
Post‐transplant lymphoproliferative disease with small bowel obstruction	**1**
Opportunistic infection:	
*Streptococcus anginosis* bacteraemia	**1**
Treatment‐resistant viral warts	**1**
Treatment‐resistant Herpes Simplex 1 mouth ulcers	**1**
Chronic ascites	**1**
De novo autoimmune hepatitis	**1**
Chronic telogen effluvium	**1**
Anastomotic stricture of hepaticojejunostomy with recurrent cholangitis	**1**

^†^ Some patients had more than one complication recorded.

There were two deaths within the cohort: (i) a patient with Crigler–Najjar syndrome who developed post‐transplant lymphoproliferative disorder; and (ii) a patient with congenital hepatic fibrosis who was subsequently reclassified as Caroli syndrome in adult services (non‐transplanted) and who suffered from a cerebral haemorrhage on a background of chronic renal failure and who developed acute respiratory failure secondary to *Klebsiella* pneumonia; both patients died in their 30s. Overall mortality in the cohort was 3%.

## Discussion

In this heterogeneous, but representative, population‐based regional cohort study of patients with paediatric‐onset liver disease, we have clearly demonstrated the high health‐care utilisation that these patients face in early adulthood. Specifically, we have shown the diverse number of individual diagnoses (27 in total) but the significant prevalence of transplantation at transfer (41%). Our data on TFS (54%) are novel for this heterogeneous group of patients and is in‐line with recent data with regard to biliary atresia.^12^ We have further highlighted basic pregnancy outcomes, potential mental health issues and transplant‐related complications faced by these patients.

The strengths of this study are the prospective nature of the original cohort, the long follow‐up duration (median > 27 years), the strict geographical nature of the cohort (highlighting transition prevalence) and the broad overview of health‐related outcomes. A broad and non‐systematic review of the current literature on PubMed (using the search terms: ((‘Follow‐Up Studies’[Mesh]) OR ‘Survival Analysis’[Mesh]) AND (‘Liver Diseases’[Mesh]) AND (‘Child’[Mesh] OR ‘Child, Pre‐school’[Mesh])) produced no regional cohort studies published in the last decade outlining the natural history of this heterogeneous group of patients. Although there are a significant number of studies evaluating single conditions (e.g. Wilson's disease^6^), and most notably in the field of liver transplantation,^13^ these studies are often not generalisable to those working in non‐transplant centres where most patients will be cared for and transitioned from.

There are of course limitations to this retrospective study. As mentioned above, phenotypic data were lacking for certain patient groups (e.g. viral hepatitis) and a detailed analysis of liver function in certain scenarios (such as pregnancy) was not available. We however feel that individual alterations in liver function are multifactorial and this would not have been a useful analysis to perform. Data on medication adherence was also not available as this is not possible (or indeed accurate) in this retrospective design. Although covering a quarter of the Scottish population, the relative rarity of paediatric‐onset chronic liver disease also means this patient cohort remained relatively small and conducting similar studies in other regional centres may be useful. It is also of interest that, again likely due to the time frame of the study, only one patient had a diagnosis of NAFLD. Given that there was almost no published research in the field of paediatric NAFLD prior to 2008, and with these patients possibly looked after in general paediatric or endocrinology clinics in the past, it is likely that future data will comprise a much larger proportion of these patients.

Health‐care professionals in adult services have the potential to encounter treatment‐related complications soon after transition compared to chronic liver diseases of adult‐onset.^14^ Within this cohort, a significant number of patients had already experienced transplant‐related complications, two of these requiring a second transplant. Burra et al. suggested that younger age at transplant is associated with increased risk of graft loss and that paediatric transplant recipients are more likely to experience immunosuppressant‐related renal dysfunction necessitating a kidney transplant.^15^ Similarly, Ferrarese et al. showed that chronic rejection and non‐adherence were prevalent (18% and 25%, respectively).^14^ However, overall, it is reassuring that children transitioning to adult services have been demonstrated to have reasonable long‐term outcome with one study showing 10‐year graft survival of 86%.^16^

Structured transition to adult services is now being seen as a crucial process and normal standard of care in patients with chronic disease,^17^, ^18^ with guidance in the field of chronic liver disease having only recently being published.^5^, ^19^ Best practice is now being established across the field and it is becoming clear that ensuring consistent MDT support, recognition of disease‐specific issues, exemplary communication between paediatric and adult teams and ongoing training and education of health‐care professionals is vital to transition success.^5^

As patients move from a more paternalistic and interdisciplinary approach to a more independent and autonomous practice in adult care,^20^ evidence indicates that there is an increase in non‐adherence as this transition occurs.^16^, ^21^ Several studies have demonstrated that a third of adolescents had been non‐adherent to clinic appointments and medications, with a significant association between older age and non‐adherence among young adults and adolescents.^15^, ^22^, ^23^, ^24^, ^25^ Preparing an adolescent for self‐management is vital,^26^ a notion further supported by previous data showing that clinic attendance in paediatrics may be a measure of parental adherence rather than the adolescent's.^22^ Therefore, it is essential to empower all patients with chronic liver disease to be proactive and begin self‐management of their health, independent of parents, through a developmentally appropriate transition process.^3^, ^20^, ^22^, ^23^, ^24^, ^25^Reproductive health consequences in this group of patients are an important aspect of their care. While this study has only reported basic information regarding pregnancies in females, fertility and offspring‐related issues also need to be considered in male patients. Currently, data on pregnancy in patients with established chronic liver disease is relatively poorly studied, with data on those diagnosed in childhood even more scarce.^27^ Many liver transplant recipients are able to go through pregnancy without major complications; however, the risk of teratogenicity with drugs such as mycophenolate mofetil or ribavirin is very real. Therefore pre‐conception planning paramount to ensure high‐risk treatments are stopped if possible to minimise pregnancy complications. Similarly, portal hypertension is ideally aggressively managed in patients with cirrhosis prior to pregnancy due to the risk of deterioration resulting in variceal bleeding.^1^, ^27^ All patients (both male and female) should therefore be encouraged to seek medical advice when they become sexually active to improve maternal and fetal outcomes.^28^

## Conclusion

Using a population‐based cohort study design, we have demonstrated the heterogeneous nature of paediatric‐onset chronic liver disease and health‐care issues encountered following handover to adult services. Further population‐based longitudinal cohort studies are required to further evaluate this group of patients to ensure that a more personalised transition programme can be adopted to improve long‐term follow‐up and reduce morbidity and mortality.
